# Grey matter correlates of affective and somatic symptoms of premenstrual dysphoric disorder

**DOI:** 10.1038/s41598-022-07109-3

**Published:** 2022-04-09

**Authors:** Manon Dubol, Johan Wikström, Rupert Lanzenberger, C. Neill Epperson, Inger Sundström-Poromaa, Erika Comasco

**Affiliations:** 1 Department of Neuroscience, Science for Life Laboratory, Uppsala University, POB 593, 75124 Uppsala, Sweden; 2 Department of Surgical Sciences, Neuroradiology, Uppsala University, Uppsala, Sweden; 3 Department of Psychiatry and Psychotherapy, Medical University of Vienna, Vienna, Austria; 4 Department of Psychiatry, Department of Family Medicine, University of Colorado School of Medicine-Anschutz Medical Campus, Aurora, USA; 5 Department of Women’s and Children’s Health, Uppsala University, Uppsala, Sweden

**Keywords:** Neuroscience, Medical research

## Abstract

Ovarian hormones fluctuations across the menstrual cycle are experienced by about 58% of women in their fertile age. Maladaptive brain sensitivity to these changes likely leads to the severe psychological, cognitive, and physical symptoms repeatedly experienced by women with Premenstrual Dysphoric Disorder (PMDD) during the late luteal phase of the menstrual cycle. However, the neuroanatomical correlates of these symptoms are unknown. The relationship between grey matter structure and PMDD symptom severity was delineated using structural magnetic resonance imaging during the late luteal phase of fifty-one women diagnosed with PMDD, combined with Voxel- and Surface-Based Morphometry, as well as subcortical volumetric analyses. A negative correlation was found between depression-related symptoms and grey matter volume of the bilateral amygdala. Moreover, the severity of affective and somatic PMDD symptoms correlated with cortical thickness, gyrification, sulcal depth, and complexity metrics, particularly in the prefrontal, cingulate, and parahippocampal gyri. The present findings provide the first evidence of grey matter morphological characteristics associated with PMDD symptomatology in brain regions expressing ovarian hormone receptors and of relevance to cognitive-affective functions, thus potentially having important implications for understanding how structural brain characteristics relate to PMDD symptomatology.

## Introduction

Specific to women’s mental health, premenstrual dysphoric disorder (PMDD) is a mood disorder characterized by psychological (i.e. affective lability, irritability, depressed mood and anxiety), cognitive (i.e. difficulties concentrating), and physical (i.e. breast tenderness, feeling bloated, musculoskeletal pain) symptoms repeatedly occuring in the late luteal phase of the menstrual cycle^1,2^. It is estimated that 3–8% of women of reproductive age meet the criteria for PMDD as delineated by the Diagnostic and Statistical Manual of mental disorders (DSM)^3^. The severity of both psychological and physical PMDD symptoms interferes with the woman's life (family, social, and work functioning), although the affective symptoms lead to greater impairment compared to physical symptoms^4^. As there is no evidence for increased or decreased ovarian hormone levels in women with PMDD, symptoms have been hypothesized to arise from maladaptive brain response to the ovarian hormone fluctuations^5^. Yet, the neural correlates of PMDD symptoms remain poorly understood. Recent neuroimaging findings suggest that the ovarian hormone fluctuations throughout the menstrual cycle influence brain structure in healthy women^6,7^. Thus, luteal phase-specific maladaptive structural responses to ovarian hormones fluctuations represent putative risk factors for PMDD. To date, brain surface correlates of PMDD symptomatology have not been investigated, and only one study investigated the grey matter volume (GMV) correlates of PMDD symptom severity, reporting negative results^8^. However, this study included a small sample of women with PMDD (n = 15) scanned across the entire luteal phase, likely covering asymptomatic time points, and important confounding factors such as total brain volume and body mass index (BMI) were not taken into account^8^. Furthermore, one study on healthy naturally cycling women suggests that regional GMV relates to subclinical premenstrual symptoms^9^. Hence, a comprehensive examination of the relationship between brain morphological measures and PMDD symptoms is missing. In line with recent evidence promoting the use of multimodal neuroimaging approaches in psychiatry research^10^, the combination of local voxel-wise measures of GMV with measures of cortical thickness and folding (i.e. cortical thickness, gyrification, sulcal depth and complexity) was here employed to yield a comprehensive analysis of grey matter structure in relation to PMDD symptomatology. The present study aimed at determining the relevance of GMV and surface morphology to PMDD symptomatology, by use of multiscale structural MRI (sMRI) analyses (i.e. voxel-based (VBM) and surface-based brain (SBM) morphometry and subcortical volumetric analyses) based on macroanatomical characteristics of cortical and subcortical grey matter. We report, for the first time, the brain structural signatures of PMDD symptoms assessed with sMRI by use of a whole-brain, automated neuroimaging analysis of VBM and SBM, complemented by cortical and subcortical region-of-interest (ROI) analyses, in a relatively large and finely characterized sample of women with PMDD.

## Results

**Figure 1:**
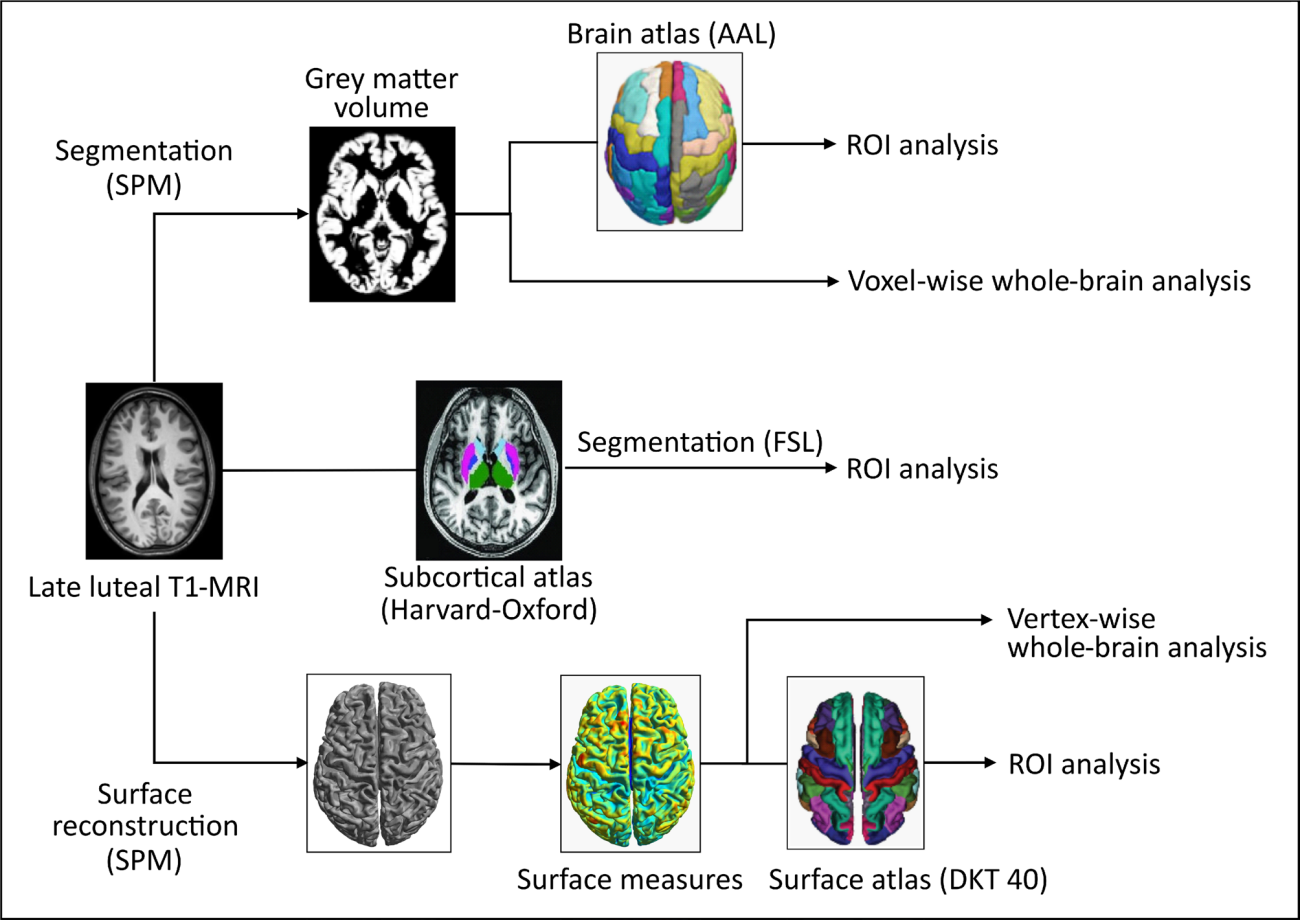
Brain structural analysis flowchart. The Surface-Based Morphometry (SBM) processing pipeline yielded four measures of surface morphology, namely cortical thickness, gyrification index, sulcal depth and cortical complexity. These four measures were used for whole-brain vertex-wise analyses, and mean values within ROIs were extracted for further analyses. The Voxel-Based Morphometry (VBM) pipeline produced grey matter probability maps that were taken onto voxel-based analyses at the whole-brain level. Subsequent ROI analyses were performed on the mean grey matter volumes within ROIs. A subcortical segmentation pipeline provided ROI measures for the amygdala and the hippocampus. *AAL* Automated Anatomical Labeling atlas, *DKT 40* Desikan–Killiany–Tourville atlas, *FSL* FMRIB Software Library, *ROI* region of interest, *SPM* Statistical Parametric Mapping.

In this cross-sectional study that prospectively tracked PMDD symptoms on a daily basis, high-resolution T1 images were collected during the late luteal phase, and sMRI analyses (VBM, SBM, and subcortical volumetric analysis) were performed (Fig. 1). Fifty-one women with PMDD (22–46 years-old) were assessed (Table 1), all having a regular menstrual cycle and 41.2% being nulliparous. On average, they were mostly right-handed (94.1%), highly educated (74.5%) and employed (84.3%). Following two diagnostic menstrual cycles (DRSP scores presented in Table S1), PMDD symptoms of mild to moderate severity were reported in the late luteal phase of the scan month (Table 2).

**Table 1:** Participants characteristics.

Demographics and psychometrics	Mean ± SD or n (%)
Sample size (n)	51 (100%)
Age (years)	34.7 ± 6.0
BMI	23.7 ± 3.4
Menarche (years)	13 ± 2
Menstrual cycle length (days)	28 ± 2
**Nicotine users**	11 (21.6%)
Smoking cigarettes	6 (54.5%)
Dipping tobacco	6 (54.5%)
Alcohol use (AUDIT score)	3.35 ± 2.28
**PMDD clinical characteristics**	
Age at onset (years)	24 ± 7
Illness duration (years)	11 ± 7
Total DRSP score	70.6 ± 15.0
Previous PMDD treatment	41 (80.4%)
SSRI	29 (70.7%)
Hormonal treatment	21 (51.2%)
Homeopathy	9 (22.0%)
Psychological support	4 (9.8%)
Psychiatric history	22 (43.1%)
Depressive disorder	17 (77.3%)
Anxiety disorder	5 (22.7%)
Depressive and anxiety disorders	3 (13.6%)
Anorexia	2 (9.1%)
Bulimia	2 (9.1%)
**Hormonal concentrations**	
Estradiol (pmol/L)	389.7 ± 239.3
Progesterone (nmol/L)	21.2 ± 16.1

*AUDIT* Alcohol use disorders test, *BMI* Body Mass Index, *DRSP* Daily record of severity of problems, *SD* Standard deviation, *SSRI* Selective serotonin reuptake inhibitor.

**Table 2:** Premenstrual symptom severity within the scan month in women with PMDD.

	DSM-V domain	DSM-V domain abbreviation	DRSP item	Mean ± SD per DRSP item	Mean ± SD per DSM domain
Core symptoms	Marked affective lability	AFFECTIVE LABILITY	Had mood swings	3.6 ± 1.4	6.9 ± 2.5
			Was more sensitive to rejection or easily hurt	3.2 ± 1.3	
	Marked irritability or anger	IRRITABILITY	Felt angry, irritable	3.7 ± 1.4	6.7 ± 2.6
			Had conflicts or problems with people	3.0 ± 1.3	
	Markedly depressed mood	DEPRESSION	Felt depressed, sad, “down” or blue	3.2 ± 1.2	9.3 ± 3.2
			Felt hopeless	3.0 ± 1.1	
			Felt worthless or guilty	3.0 ± 1.1	
	Marked anxiety and tension	ANXIETY	Felt anxious, “keyed up” or “on edge”	3.2 ± 1.3	
Secondary symptoms	Decreased interest in usual activities	ANHEDONIA	Had less interest in usual activities	3.3 ± 1.2	
	Difficulty in concentration	CONCENTRATION	Had difficulty concentrating	3.2 ± 1.3	
	Lethargy and marked lack of energy	ENERGY LOSS	Felt lethargic, tired, fatigued, or had a lack of energy	3.6 ± 1.2	
	Marked change in appetite	APPETITE	Had increased appetite or overate	2.5 ± 1.4	5.1 ± 2.6
			Had specific food craving	2.6 ± 1.4	
	Hypersomnia or insomnia	SLEEP	Slept more, tool naps, found it hard to get up	3.0 ± 1.1	5.7 ± 2.2
			Had trouble getting to Sleep, staying asleep	2.7 ± 1.5	
	Feeling overwhelmed or out of control	OVERWHELMED	Felt overwhelmed, that I couldn’t cope	3.1 ± 1.2	5.8 ± 2.2
			Felt out of control	2.7 ± 1.2	
	Physical symptoms	PHYSICAL	Had breast tenderness	2.4 ± 1.5	7.4 ± 3.4
			Had breast swelling, felt bloated, or had gain weight	2.9 ± 1.5	
			Had joint or muscle pain	2.1 ± 1.2	
	–		Had headache	2.1 ± 1.2	–
	Total DRSP score			62.3 ± 18.4	

The mean late luteal phase DRSP scores for individual items were obtained during the final five days of the menstrual cycle in the scan month. In addition, the mean late luteal phase total DRSP score, and DRSP subscales corresponding to the DSM-V domains were computed by summing the individual DRSP items. *DSM* Diagnostic and Statistical Manual of Mental Disorders, *DRSP* Daily Record of Severity of Problems, *SD* standard deviation.

### Whole-brain grey matter correlates of PMDD symptoms

**Figure 2:**
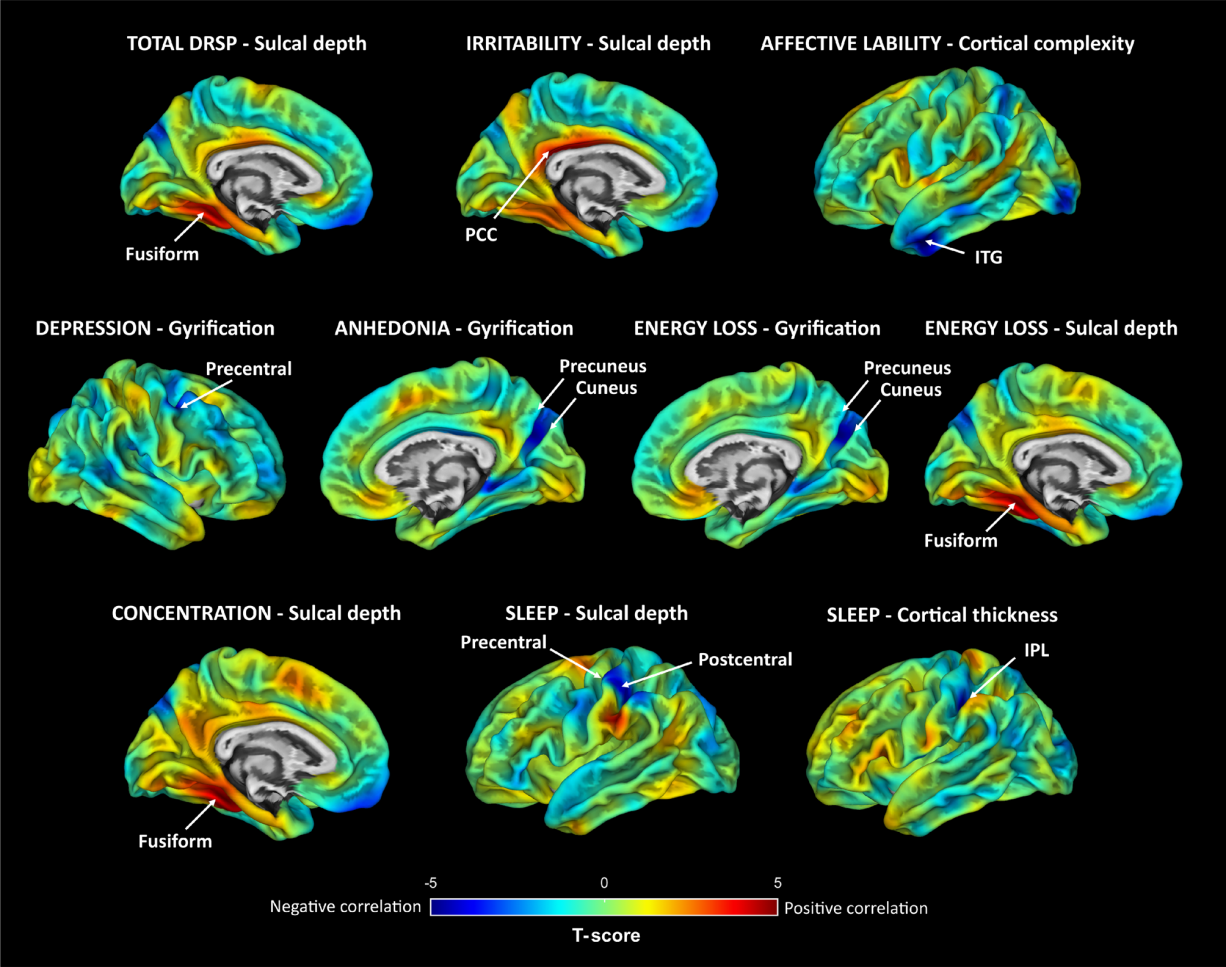
Whole-brain associations between surface measures and PMDD symptom severity. Results from partial correlation analyses ran to assess the relationship between brain surface measures and DRSP scores corresponding to PMDD symptoms as defined by the DSM-V domains. Unthresholded SPM T-maps are overlaid on an average central surface. Regions displaying significant (p_FWE_ < 0.05) correlations with PMDD symptom severity are indicated by arrows. Positive and negative correlations between surface measures and DRSP scores are illustrated in red and blue, respectively. Findings not reaching statistical significance (p_FWE_ < 0.05) are not represented. *DRSP* Daily Record of Severity of Problems, *Fusiform* Fusiform gyrus, *IPL* inferior parietal lobule, *ITG* inferior temporal gyrus, *PCC* posterior cingulate cortex, *Precentral* precentral gyrus, *Postcentral* postcentral gyrus.

Associations between surface measures and Daily Record of Severity of Problems (DRSP) scores obtained from the whole-brain vertex-wise analyses are presented in Fig. 2 and Table S2 (a detailed description is provided as supplementary information). Among the whole-brain results, correlation coefficients ranged from 0.48 to 0.58 (95% CI [0.23–0.73]), indicating moderate correlations. The most significant findings (p_FWE_ < 0.05 corrected for the number of voxels and tests) illustrate positive correlations between sulcal depth measures in the left fusiform gyrus (FuG) and the scores of the total DRSP, concentration and energy loss, as well as the score of irritability in the left posterior cingulate cortex (PCC). At the whole-brain level, no significant associations between GMV and PMDD symptom severity were detected (p > 0.05 FWE corrected).

### ROI-based grey matter correlates of PMDD symptoms

**Figure 3:**
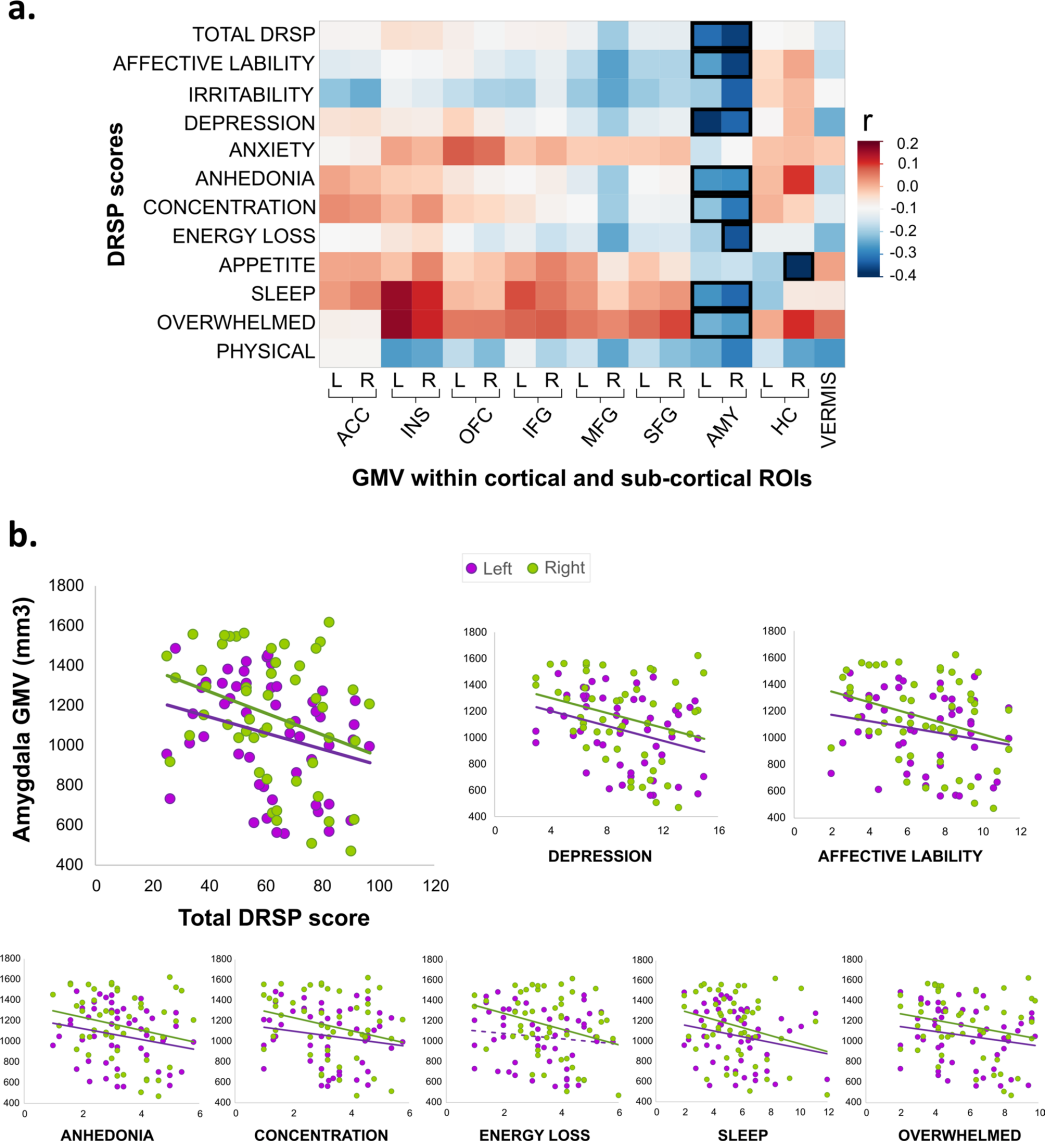
Correlation of GMV within ROI by PMDD symptom severity. (**a**) Correlation between DRSP scores corresponding to PMDD symptoms as defined by the DSM-V domains, and raw grey matter volume (GMV) within the cortical, and subcortical ROIs. The colors represent the correlation coefficient values. Positive and negative correlations are illustrated in red and blue respectively. **⎕**, p < 0.05 after correcting for TIV, age and BMI. Rectangles including left and right ROIs indicate significant correlations obtained for the bilateral average ROI. The correlation heatmap was generated using JMP10 (JMP, SAS Institute). (**b**) Scatterplots depicting the correlations between GMV of the left and right amygdala (raw values, unadjusted for TIV, age and BMI) and the total DRSP score, as well as the scores of the PMDD domains affective lability, depression, anhedonia, concentration, energy loss, sleep and overwhelmed (− 0.33 < r < − 0.29). Dotted lines indicate non-significant correlations. *ACC* anterior cingulate cortex, *AMY* amygdala, *DRSP* Daily Record of Severity of Problems, *HC* hippocampus, *INS* insula, *L* left, *MFG* middle frontal gyrus, *OFC* orbitofrontal cortex, *R* right, *SFG* superior frontal gyrus, *VERMIS* cerebellar vermis.

**Figure 4:**
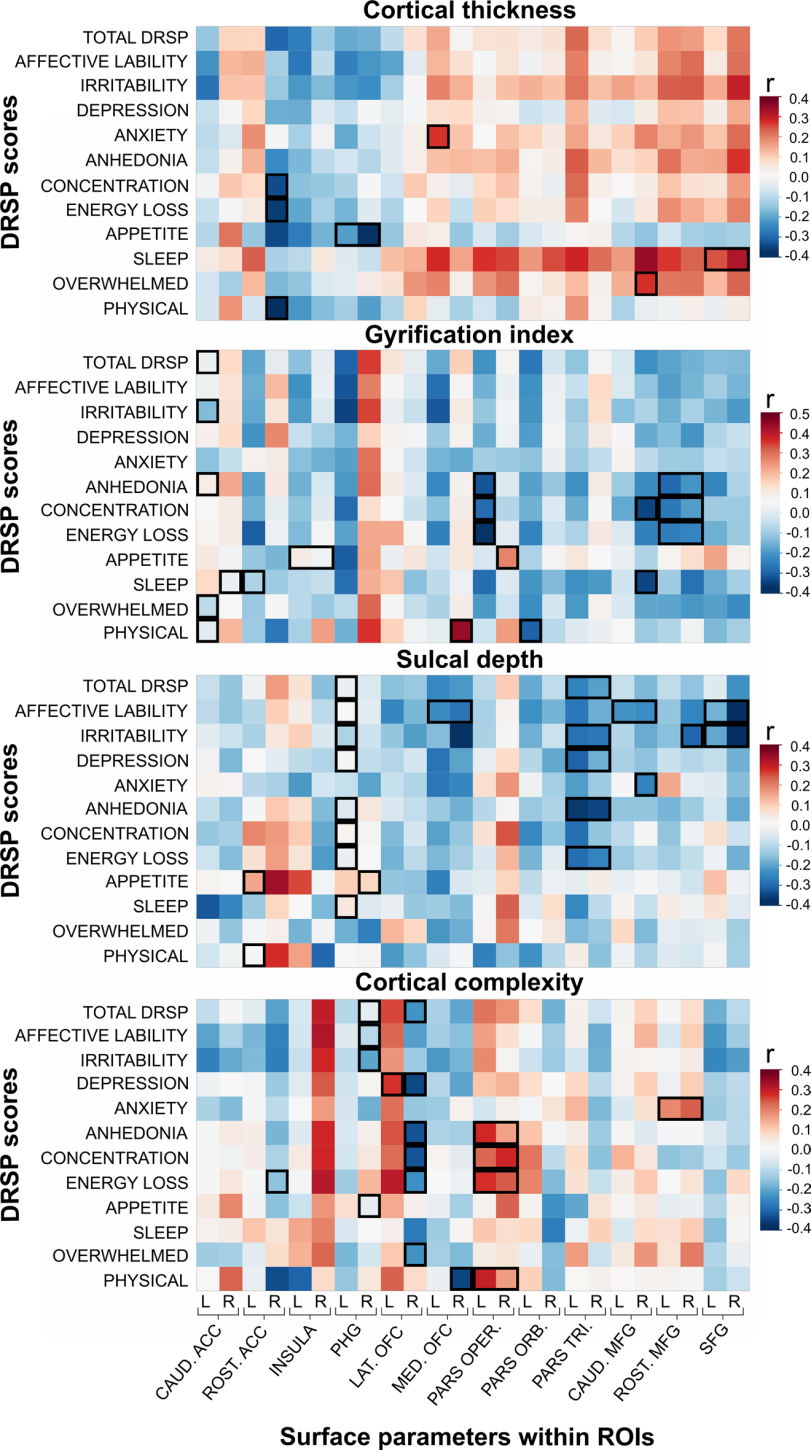
Correlation heatmaps of surface measures within ROI by PMDD symptom severity. Correlation between DRSP scores corresponding to PMDD symptoms as defined by the DSM-V domains, and surface parameters within the cortical ROIs: cortical thickness, gyrification index, sulcal depth, and cortical complexity. The colors represent the correlation coefficient values. Positive and negative correlations are illustrated in red and blue respectively. **⎕**, p < 0.05 after correcting for TIV, age and BMI. Rectangles including left and right ROIs indicate significant correlations obtained for the bilateral average ROI. The correlation heatmaps were generated using JMP10 (JMP, SAS Institute). *ACC* anterior cingulate cortex, *CAUD* caudal, *DRSP* Daily Record of Severity of Problems, *L* left, *LAT* lateral, *MED* medial, *MFG* middle frontal gyrus, *OFC* orbitofrontal cortex, *PARS ORB* pars orbitalis, *PARS OPER* pars opercularis, *PARS TRI* pars triangularis, *PHG* parahippocampal gyrus, *R* right, *ROST* rostral, *SFG* superior frontal gyrus.

Complementary to whole-brain explorative analyses, ROI analyses were conducted to investigate the relationship between PMDD symptoms and a priori defined brain structures, in line with previous neuroimaging findings on PMDD^5^. Relationships between DRSP scores and measures of GMV extracted from cortical ROIs (prefrontal cortex (PFC), anterior cingulate cortex (ACC), cerebellum vermis, parahippocampal gyrus (PHG), and insula) and from subcortical finer segmentation of deep structures (amygdala, hippocampus) were investigated. Among the ROI results, correlation coefficients ranged from 0.27 (95% CI [− 0.01–0.50]) to 0.43 (95% CI [0.17–0.63]), indicating moderate correlations (Figs. 3 and 4). Among these findings, the strongest correlations primarily point to relationships between the severity of various PMDD symptoms and amygdalar volume (Table S3), as well as surface measures of prefrontal, anterior cingulate and parahippocampal regions (Table S4). A detailed description is provided as supplementary information.

## Discussion

We report on neuroanatomical correlates of symptom severity in women with PMDD, for the first time investigated through a combined whole-brain and ROI multi-scale automated MR assessment ensuring high morphological accuracy. By taking advantage of the fact that PMDD symptoms may vary in intensity from cycle to cycle, we found, in women already diagnosed with PMDD, that current variations in the severity of PMDD symptoms were associated with surface measures, particularly in the prefrontal, cingulate and parahippocampal areas, but also in insular, temporal, parietal, occipital and paracentral regions. Surface measures of the caudal ACC, PHG, inferior-orbital PFC regions and FuG seemed particularly involved in PMDD symptomatology, as they displayed correlations with the total DRSP score (Fig. S1). Additionally, PMDD symptom severity was associated to GMV of the amygdala. The present findings have implications for understanding how morphological brain characteristics relate to PMDD symptomatology.

The localization of the surface correlates of PMDD symptoms overlaps with brain regions whose fluctuations of GMV have been associated with the menstrual cycle in healthy women, such as the PHG, ACC, PFC and FuG^7^; all regions characterized by expression of ovarian hormone receptors^11,12^ and of relevance to cognitive-affective functions^13^. In addition, our findings relate to the Default Mode Network, where the PHG, ACC, PCC, precuneus, and medial PFC areas constitute anatomical and functional hubs^14,15^. In healthy women, menstrual cycle-related variations of functional connectivity were reported between regions of this network^7^, whose activity has been associated with depression^16^. Moreover, the functional connectivity of the PHG with the PCC has been associated with negative mood in patients with remitted depression^17^. Consistently, our results point towards associations between the affective core PMDD symptoms and the surface measures of the PHG (affective lability, irritability and depression) and PCC (irritability). Furthermore, among the numerous correlations we found between PMDD symptom severity and surface parameters within frontal areas, most involved anterior cingulate and ventrolateral-orbital areas, although associations were also observed in ventromedial and dorsal areas, to a lesser extent. This is of particular interest, as the ACC and ventrolateral PFC are primarily involved in cognitive control, most commonly recruited during emotion regulation^18^. Corroborating findings from functional neuroimaging studies particularly highlight the implication of these regions in PMDD (e.g. blunted fronto-cingulate activation in PMDD patients while processing emotions^19^). To date, the sole SBM study comparing women with PMDD and healthy controls reports no differences in cortical thickness over the whole brain^20^. Thus, the present findings represent the first SBM analysis to provide evidence of associations between cortical morphology and the severity of PMDD symptoms, although causality could not be determined. Moreover, lateralized patterns of correlations between surface measures and symptom severity emerged from our findings, particularly in the ACC, PHG and orbitofrontal cortex (OFC). However, the asymmetry index of ROIs indicates minor differences between the structural measures of the two hemispheres (Fig. S2), thus suggesting that PMDD symptoms arise from region- and hemisphere-specific function.

Complementary to surface results, the present volumetric findings show correlations between the PMDD symptom severity and subcortical GMV. Negative correlations were observed between GMV of the bilateral amygdala and the severity of affective core and secondary PMDD symptoms; which are all related to the depressive symptomatology^1^. The amygdala is thought to play a role in depression, and is central to the hypothesized impairment of top-down inhibitory processes in PMDD^5^. Indeed, the functional MRI literature points to concurrent hyperactivity of the amygdala^21^ and hypoactivity of dorsolateral prefrontal areas^22^, both associated with PMDD symptom severity in the late luteal phase. Anatomical compensatory effects, or vice versa predisposition in terms of small regional volume, could be hypothesized to explain the hormonally-triggered exaggerated amygdalar functioning observed in PMDD^5^. On the cellular level, in female rodents, ovarian hormones are involved in rapid fluctuations of dendritic density in the amygdala across the estrous cycle^23–25^, as well as in depression- and anxiety-like behavior. Thus, decreasing hormones levels during the premenstrual phase could lead to decreasing GMV in this region and increased symptom severity in women with PMDD.

The observed correlations between psychological symptom severity and grey matter measures were located within the corticolimbic circuit, primarily in areas of relevance to interlinked cognitive and emotion processing. The findings involve limbic (amygdala), paralimbic (parahippocampus), and higher-order integrative cortical areas such as the PFC and ACC, known to be implicated in anxiety and depression^26,27^. Thus, together with the albeit limited neuroimaging findings on PMDD^5^ and studies on mood disorders and healthy samples, the present results have important implications for understanding the neuroanatomical correlates of symptoms overlapping over diagnostic categories, in line with the dimensional approach promoted by the Research Domain Criteria project^28^.

Moreover, the present study identifies for the first time the neuroanatomical correlates of premenstrual somatic symptoms, which overlap with a neural network implicated in somatoform disorders involving structures mediating visceral-somatic perception, emotional processing, and cognitive control, such as the ACC, OFC, insula, hippocampal formation, amygdala and dorsolateral PFC^29^. Interestingly, it has been shown that somatization is involved in the severity of premenstrual symptoms in healthy women^30^. Further, it has been proposed that the structure and function of the emotional brain plays a critical role in linking nociception and pain perception, in light of the interactions between anxiety, depression and pain^31^. Remarkably, the regions where we found associations between surface measures and physical symptoms overlap with the ones for which we found associations with affective symptoms, namely the ACC, medial OFC, and inferior frontal gyrus (IFG) sub-regions. A symptoms-based discussion of the findings is presented as supplementary information.

In view of that PMDD symptom severity fluctuates as a main characteristic of the disorder, the present findings could suggest that the PMDD brain undergoes menstrual cycle-phase dependent structural changes in the neural network subserving corticolimbic and related association areas, thus representing maladaptive neuroanatomical response to hormonal fluctuations^5^. In line, the present neuroanatomical correlates of PMDD symptoms seem to differ from the anatomical signatures of PMDD, in comparison with healthy controls, which have been sparsely investigated by use of VBM and SBM analyses in four studies and yield conflicting results^5^. While anatomical fluctuations occur in the brain of healthy naturally cycling women throughout the menstrual cycle; the late luteal phase remains virtually unstudied in healthy women^7^. Correlates of emotion processing, cognition, brain metabolism and neurotransmission point to differential menstrual cycle-related neuroadaptive changes in women with PMDD compared to healthy naturally cycling women^5^, involving structures such as the PFC, insula, cerebellum, and amygdala, which overlap with the regions described as being influenced by the menstrual cycle in healthy women^6,7^ and the ones here indicated as correlates of PMDD symptom severity.

The present findings should be interpreted in light of the following methodological considerations. The current study displays a number of strengths compared to the neuroimaging literature on PMDD, such as the use of multi-scale, highly standardized, structural brain analyses and a rather large and well-characterized sample of women with PMDD. Furthermore, confirmation of menstrual cycle phase was ensured through both menstrual cycle mapping and hormonal assessment, and both age and BMI were considered as potential confounding factors influencing brain structure. Therefore, this study offers greater statistical power compared to previous work on PMDD. Thus, while Jeong et al.^8^ exclusively performed whole-brain voxel-wise analyses of GMV, we combined whole-brain investigations with a ROI approach providing increased sensitivity to the neuroimaging analyses by reducing the number of comparisons that need to be controlled for. In addition, we conducted subcortical segmentation of the hippocampus and amygdala, in order to circumvent the low contrast between tissues within subcortical structures in MR images^32^. Of note, whole-brain analyses of GMV conducted in our sample of women with PMDD did not reveal any significant correlation with symptom severity, in accordance with Jeong et al.^8^. Furthermore, although VBM findings might be driven by variability in cortical thickness and/or folding^33^, SBM provides complementary measures of cortical anatomy. Nevertheless, while the use of complementary assessments (i.e. VBM, SBM, subcortical segmentation) provides a multiscale overview of the structural variations that could relate to PMDD symptoms in the brain, some ROIs could not be accurately evaluated using a combination of these methods. Furthermore, although several of the ROI-based findings did not reach significance after correction for multiple testing, the observed correlation strength up to r = 0.43 indicates substantial associations between grey matter structure and PMDD symptoms. With a sample size of fifty-one women included in the analyses, our volumetric findings reached a statistical power up to 0.82 (r = 0.43), while the surface-based results reached a power up to 0.99 (r = 0.58). Nevertheless, in order to detect effects smaller than r = 0.38 at α = 0.05 with a statistical power of at least 0.80, a larger sample of participants would be needed^34^. In addition, as only women with PMDD were assessed in this study, we cannot exclude the possibility that the associations we found between grey matter structure and premenstrual symptoms also appear in healthy women experiencing premenstrual symptoms. Last, in future studies, it would be interesting to explore the relationship between PMDD symptoms severity and brain measures in the late luteal phase compared with the asymptomatic phase, as whether the present findings represent long lasting changes of the brain organization or late luteal-specific neuroplasticity is still to be determined.

The present findings point to multi-scale neuroanatomical correlates of symptom severity in PMDD patients, investigated through a combined whole-brain and ROI automated MR data analysis approach. Variations in the severity of PMDD symptoms were associated with the volume of the amygdala, as well as surface measures of prefrontal, cingulate, temporal, parietal, occipital and paracentral regions. The present findings move forward the field by addressing a gap, demonstrating that brain morphological characteristics are related with PMDD symptomatology and potentially have important implications for understanding differential brain function previously associated with PMDD.

## Materials and methods

### Participants

This study was carried out at the Departments of Obstetrics and Gynecology at Uppsala University Hospital, from 2016 to 2019. Sixty-two women with PMDD (22–46 years) with regular menstrual cycles (25–35 days), of Caucasian origin and Swedish-speaking were recruited by advertisement in local newspapers, boards, social media, and students’ websites. Exclusion criteria were: steroid hormone treatment during the previous three months (including hormonal contraceptives), breast-feeding, pregnancy, presence of ongoing psychiatric disorders, treatment with psychotropic drugs during the previous three months, severe medical conditions, and contraindications for MRI. All procedures were conducted in compliance to the Declaration of Helsinki and approved by the ethic committee of Uppsala (Dnr. 2016/184 and 2016/312). Written informed consent was obtained from all participants.

PMDD diagnosis according to DSM-5 criteria was confirmed using daily prospective symptom ratings during two consecutive menstrual cycles with the Daily Report Severity of Problems (DRSP) scale (Table S1) using a smartphone application. Thus, the participants were required to present marked symptoms in the luteal phase, causing significant distress or interference with usual activities, to meet the PMDD diagnosis criteria. PMDD was defined as > 50% increase in at least five of eleven symptoms (among which at least one symptom was a core PMDD symptom) between the follicular (day 6 to 12) and luteal phase (day − 7 to − 1). Percent increase was calculated as [((mean luteal phase scores – mean follicular phase scores)/mean follicular phase scores) × 100]. In addition, we required that diagnostic symptoms be at least mild (mean luteal phase score > 3.0; at least two days with scores ≥ 4) and disappeared during the follicular phase (mean follicular phase score < 2.0). Other psychiatric disorders were ruled out by the Mini-International Neuropsychiatric Interview^35^.

Primary measures of symptom severity included the mean late luteal phase total DRSP score obtained during the final 5 days of the scan month^36^. In addition, DRSP subscales were computed according to the DSM-5 categorization of PMDD core and secondary symptoms as described in^37^. DRSP sub-scales include core mood symptoms such as marked affective lability, marked irritability, marked depressed mood*,* anxiety*,* and secondary behavioral and somatic symptoms such as marked change in appetite, sleep, feeling overwhelmed, physical symptoms, anhedonia, problems concentrating and energy loss^37^. Monitoring of the menstrual cycle phase was confirmed by serum progesterone and estradiol concentrations. Due to technical issues (n = 3), brain tumor (n = 1), extreme BMI (n = 2), high AUDIT score (n = 1), missing DRSP data (n = 3) and poor grey matter segmentation output (n = 1), a total of eleven participants were excluded from the MR correlation analyses, which thus included fifty-one women.

### Hormone analyses

Venous blood samples were collected from each participant at the beginning of every session to determine the levels of estradiol and progesterone. Serum steroid hormones concentrations were measured from 300 µL of sample material at the Core Facility of Metabolomics, University of Bergen, by liquid chromatography—tandem mass spectrometry. An Acquity UPLC system (Waters, Milford, MA, USA) was used to chromatographically separate the steroids on a C-18 column (50 × 2.1 mm, 1.7 mm particle size). The UPLC system was connected to a Waters Xevo TQ-S tandem a mass spectrometer equipped with an electrospray ionization source, and the steroids were detected in negative (estradiol) or positive ion (progesterone) MRM mode. Analytical sensitivity and precision were determined as lower limit of detection and total coefficient of variation, respectively, for estradiol (3.6 pmol/L and 10.0%) and progesterone (0.21 nmol/L and 8.9%).

### MR acquisition

All participants were asked to have their brain scanned at rest in the late luteal phase of the menstrual cycle. Acquisition of high-resolution Magnetic Resonance (MR) imaging data was conducted with a 3.0 Tesla whole-body scanner (Achieva dStream, Philips Medical Systems, Best, The Netherlands) equipped with a 32-channel head coil. Acquisition of anatomical 3D-T1-weighted whole-brain scans was carried out using a MPRAGE sequence with the following parameters: Repetition Time (TR) = 8.3 ms, Echo Time (TE) = 3.8 ms, 256 × 256 matrix size, flip angle = 8°, 220 slices, acquisition time: 3:50 min. Resulting images have a 0.94 × 0.94 × 1 mm^3^ voxel size with a dimension of 256 × 256 × 220.

### Voxel-based morphometry

The MR preprocessing steps were run using the Statistical Parametric Mapping software (SPM12, Welcome Trust Centre for Neuroimaging, University College London, UK) implemented in MATLAB R2018a (MathWorks, Natick, MA, USA). First, all images were manually reoriented using the coordinate of the anterior commissure as origin (0, 0, 0), so that the orientation approximated MNI (Montreal Neurological Institute) space. Using the segment routine of SPM 12, the reoriented images were spatially normalized into the MNI space and corrected for intensity variations before being segmented into grey matter, white matter, cerebrospinal fluid, bone, soft tissue and background probability maps based on voxel intensities of MNI tissue probability maps^38^. Following segmentation, a modulation process was applied to the grey matter and white matter probability maps in order to compensate for the effects of spatial normalization on volumetric data. Finally, modulated grey matter probability maps were smoothed using an 8-mm full-width half-maximum (FWHM) Gaussian kernel, resulting in a 1.5 × 1.5 × 1.5 mm^3^ voxel size.

A quality assessment procedure including a visual inspection for apparent artefacts and an automated quality control using the CAT12 toolbox in SPM (http://dbm.neuro.uni-jena.de/vbm/check-sample-homogeneity) has been followed in order to detect image artefacts and anatomical outliers. Resulting from brain segmentation, the average total GMV was 0.73 ± 0.05 L, the average total white matter volume was 0.41 ± 0.04 L and the total cerebrospinal fluid was 0.33 ± 0.06 L. The mean Total Intracranial Volume (TIV) was 1.47 ± 0.09 L.

### Subcortical segmentation

In order to circumvent the low contrast between tissues within subcortical structures in MR images^32^ affecting the accuracy of whole-brain segmentation pipelines, and obtain reliable estimations of subcortical regional volumes, we used the FSL-FIRST segmentation pipeline through the “run_first_all” script using default settings (FSL version 6.0.0.). FSL-FIRST is based on a Bayesian probabilistic model relying on shape and intensity profiles to determine the location of subcortical structures, trained on 336 manually-labelled T1-weighted MR images. A full description of the pipeline is available in^39^. In brief, a two-stage affine registration to MNI space is applied, to the whole brain first and to subcortical regions secondarily. The segmentation process extracts each subcortical structure according to the model, based on shape and intensity. Last, a boundary correction is applied to the segmented images to classify the boundary voxels as belonging to the structure or not and to correct for overlapping segmentations. FSLstats tool was used to estimate the mean volumes of the segmented left and right amygdala and hippocampus. The method has been shown to give accurate and robust results for the segmentation of subcortical structures, comparable to—or better than—other automated methods of subcortical segmentation^39^, showing for example the least overestimation of hippocampal volumes^40^.

### Surface-based morphometry

For SBM preprocessing and analysis, we used the automated CAT12 preprocessing pipeline (http://dbm.neuro.uni-jena.de/cat). This pipeline includes a projection-based thickness estimation that allows the computation of cortical thickness and central surface in one step^41^, along with partial volume correction and correction for sulcal blurring and sulcal asymmetries. A gyrification index was extracted based on absolute mean curvature^42^. In addition, cortical complexity and sulcal depth measures were extracted. For inter-subject comparisons, the surface meshes were re-parameterized into a common coordinate system using spherical maps^43^. Finally, all surface measures were resampled and smoothed with a Gaussian kernel, of 15 mm (FWHM) for cortical thickness and 20 mm (FWHM) for the other parameters. Following preprocessing, the automated CAT12 quality control module for surface data was used in order to exclude outliers.

### Statistical analyses

Following preprocessing, fifty-one women with PMDD were included in the analyses. In order to account for the nuisance variance of regressors of non-interest, total brain volume (TIV), BMI and age were included as confounding covariates due to their influence on volumetric and surface measures^44–46^. A flowchart illustrating MR preprocessing and morphological measures used in the statistical analyses is provided in Fig. 1.

VBM statistical analyses were carried out in SPM12. VBM exploratory analyses of grey matter probability maps consisted of voxel-wise correlation analyses conducted within an explicit mask of grey matter generated from the mean grey matter probability map of the whole sample with an absolute threshold set at 0.2. Additionally, we defined ROIs based on the results of the previous literature^5^, including bilateral ACC, PFC, insula, and cerebellum vermis. PFC ROIs were divided into the OFC (gyrus rectus and orbital parts superior, middle and inferior frontal gyri), the inferior, middle and superior frontal gyri (IFG, MFG and SFG). The seven ROIs were defined according to the AAL atlas, using the Pickatlas toolbox in SPM12. Similar to voxel-wise analyses, we performed partial correlation analyses to explore the relationship between the clinical features of women with PMDD (DRSP scores) and the mean GMV extracted from both the cortical (ACC, insula, OFC, IFG, MFG, SFG and cerebellum vermis) and subcortical ROIs (amygdala and hippocampus).

SBM statistical analyses were carried out using the CAT12 toolbox in SPM12. We assessed the relationship between DRSP scores and brain surface parameters (including cortical thickness, gyrification index, cortical complexity and sulcal depth), using whole-brain vertex-wise correlation analyses. In addition, regional mean surface data were extracted using the automatic “Extract ROI-based surface values” and “Estimate mean values inside ROI” tools in CAT12, based on the FreeSurfer Desikan/Killiany atlas^47^. Twelve bilateral ROIs were analyzed based on the results of the previous literature^5^, including caudal ACC, rostral ACC, insula, PHG, and eight prefrontal regions (superior frontal, pars opercularis, pars orbitalis, pars triangularis, rostral middle frontal, caudal middle frontal, lateral orbitofrontal and medial orbitofrontal). Similar to VBM analyses, we performed partial correlation analyses to explore the relationship between ROI mean surface parameters and the clinical scores of women with PMDD.

Based on previous literature showing asymmetry in grey matter structure^48^ and brain function related to emotion regulation^49^, the ROI-based analyses were carried out for each hemisphere separately. In cases where a similar pattern of association between structural measures and symptom severity was observed for both hemispheres, we merged left and right ROIs to present the results of the average bilateral region. Statistical analyses conducted on ROI-extracted data were performed using the Statistical Package for the Social Sciences (SPSS) version 26, and the significance threshold was set at p < 0.05, uncorrected for multiple testing. According to the explorative approach of this original study, the ROI-based results are presented uncorrected for multiple testing, while indicating if significance was reached after correction [Bonferroni correction for the four affective core PMDD symptoms (p_Bonferroni_ < 0.0125), and the seven secondary symptoms (p_Bonferroni_ < 0.0071)]. Exploratory whole-brain analyses results were visualized at a significance threshold of p < 0.001 uncorrected for multiple testing, and were considered significant at p < 0.05 Family Wise Error (FWE) corrected. Trend-level results were defined as 0.05 < p < 0.1 for FWE-corrected statistics, and as 0.05 < p < 0.06 for ROI-based statistics uncorrected for multiple testing.

## Supplementary Information

Supplementary Information.

## Data Availability

The datasets generated and/or analysed during the current study are available from the corresponding author on reasonable request.
