# Supplementary material for: Grey matter correlates of affective and somatic symptoms of premenstrual dysphoric disorder
Source: Sci Rep. 2022 Apr 9;12:5996. doi: 10.1038/s41598-022-07109-3 (PMC8994757; doi:10.1038/s41598-022-07109-3)
Supplement: Supplementary file 1 — Supplementary Information. [file 41598_2022_7109_MOESM1_ESM.docx]

**Supplementary information**

Grey matter correlates of affective and somatic symptoms of premenstrual dysphoric disorder

Manon Dubol ^1^, Johan Wikström ^2^, Rupert Lanzenberger ^3^, C Neill Epperson ^4^, Inger Sundström-Poromaa ^5^, Erika Comasco ^1*^

1. Department of Neuroscience, Science for Life Laboratory, Uppsala University, Sweden;
2. Department of Surgical Sciences, Radiology, Uppsala University, Sweden;
3. Department of Psychiatry and Psychotherapy, Medical University of Vienna, Austria;
4. Department of Psychiatry, Department of Family Medicine, University of Colorado School of Medicine- Anschutz Medical Campus, USA; ^5^ Department of Women’s and Children’s Health, Uppsala University, Sweden

*Correspondence to Erika Comasco, Dept. of Neuroscience, Uppsala University BMC, POB 593, SE-75124, Uppsala, Sweden; erika.comasco@neuro.uu.se

|  | **DRSP item** | **Mean score (SD)** |
| --- | --- | --- |
| **Core symptoms** | Had mood swings | 4.1 (1.0) |
|  | Was more sensitive to rejection or easily hurt | 3.7 (0.9) |
|  | Felt angry, irritable | 4.1 (1.0) |
|  | Had conflicts or problems with people | 3.4 (1.0) |
|  | Felt depressed, sad, “down” or blue | 3.7 (1.0) |
|  | Felt hopeless | 3.5 (0.9) |
|  | Felt worthless or guilty | 3.6 (0.9) |
|  | Felt anxious, “keyed up” or “on edge” | 3.5 (0.9) |
| **Secondary symptoms** | Had less interest in usual activities | 3.7 (1.0) |
|  | Had difficulty concentrating | 3.5 (1.1) |
|  | Felt lethargic, tired, fatigued, or had a lack of energy | 4.1 (0.9) |
|  | Had increased appetite or overate | 3.0 (1.3) |
|  | Had specific food craving | 3.1 (1.2) |
|  | Slept more, tool naps, found it hard to get up | 3.4 (1.1) |
|  | Had trouble getting to Sleep, staying asleep | 3.0 (1.3) |
|  | Felt overwhelmed, that I couldn’t cope | 3.4 (1.0) |
|  | Felt out of control | 3.0 (1.0) |
|  | Had breast tenderness | 2.7 (1.5) |
|  | Had breast swelling, felt bloated, or had gain weight | 3.5 (1.5) |
|  | Had joint or muscle pain | 2.5 (1.3) |
|  | Had headache | 2.2 (1.1) |
| **Functioning** | At work, school, home, or in daily routine, at least one of the problems noted above caused reduced productivity or inefficiency | 3.7 (1.0) |
|  | At least one of the problems noted above interfered with hobbies or social activities (e.g., avoided or did less) | 3.6 (1.1) |
|  | At least one of the problems noted above interfered with relationships with others | 3.6 (1.1) |
| **Total DRSP score** | | 70.6 (15.0) |

**Table S1: Premenstrual symptom severity during the diagnostic months in women with PMDD.** The mean late luteal phase DRSP scores for individual items were obtained during the final five days of the menstrual cycle of the two prospective diagnostic months. In addition, the mean late luteal phase total DRSP score was computed by summing the individual DRSP items. The scores averaged over the two prospective diagnostic months is presented. DRSP, Daily Record of Severity of Problems; SD, standard deviation.

| **DRSP scores and items** | **Surface Measure** | **Direction of effect** | **Cluster Level** | | | **Voxel level** | | | | | | | |
| --- | --- | --- | --- | --- | --- | --- | --- | --- | --- | --- | --- | --- | --- |
|  |  |  | **Cluster Size**  **(voxels)** | **p_FWE_** | **p uncorr** | **r** | **t** | **p_FWE_** | **p uncorr** | **MNI Coordinates** | | | **Region (aal)** |
|  |  |  |  |  |  |  |  |  |  | **x** | **y** | **z** |  |
| **TOTAL DRSP** | SD | Positive ^c^ | 294 | 0.001 *^a^* | 9.0614e-04 | 0.48 | 3.83 | 0.310 | 1.9274e-04 | -35 | -37 | -18 | Fusiform_L |
| **Core symptoms** | | | | | | | | | | | | | |
| AFFECTIVE LABILITY | SD | Negative | 79 | 0.196 | 0.150 | - 0.53 | 4.51 | 0.057 | 2.2197e-05 | -4 | 55 | -20 | Rectus_L |
|  |  |  | 111 | 0.096 | 0.070 | - 0.55 | 4.36 | 0.085 | 3.5783e-05 | -59 | -2 | -26 | Temporal_Mid_L |
|  | CC | Negative ^b^ | 94 | 0.115 | 0.071 | - 0.57 | 4.84 | 0.025 | 7.1870e-06 | -47 | -3 | -35 | Temporal_Inf_L |
| IRRITABILITY | SD | Positive ^c^ | 222 | 0.007*^a^* | 0.005 | 0.48 | 3.93 | 0.249 | 1.4005e-04 | -7 | -41 | 25 | Cingulum_Post_L |
|  |  | Negative | 124 | 0.071 | 0.051 | - 0.55 | 4.55 | 0.051 | 1.9493e-05 | -59 | -3 | -27 | Temporal_Mid_L |
| DEPRESSION | GI | Negative ^b^ | 64 | 0.251 | 0.174 | - 0.54 | 4.80 | 0.029 | 8.6811e-06 | 44 | -1 | 46 | Precentral_R |
| ANXIETY | N.S. | | | | | | | | | | | | |
| **Secondary symptoms** | | | | | | | | | | | | | |
| ANHEDONIA | CT | Positive | 49 | 0.342 | 0.227 | 0.51 | 4.43 | 0.090 | 2.9147e-05 | -5 | 40 | 44 | Frontal_Sup_Medial_L |
|  | GI | Negative ^b^ | 99 | 0.104 | 0.066 | - 0.58 | 4.64 | 0.046 | 1.4503e-05 | 20 | -69 | 28 | Cuneus-Precuneus_R |
|  | SD | Negative | 58 | 0.304 | 0.252 | - 0.51 | 4.46 | 0.065 | 2.6285e-05 | 56 | 1 | 38 | Precentral_R |
| CONCENTRATION | SD | Positive ^b,c^ | 326 | 6.2676e-04*^a^* | 4.3629e-04 | 0.53 | 4.60 | 0.044 | 1.6371e-05 | -34 | -36 | -19 | Fusiform_L |
| ENERGY LOSS | SD | Positive ^c^ | 62 | 0.281 | 0.229 | 0.52 | 4.39 | 0.079 | 3.3164e-05 | -41 | -68 | 15 | Occipital_Mid_L |
|  |  |  | 449 | 3.3565e-05*^a^* | 2.3338e-05 | 0.53 | 4.27 | 0.107 | 4.7797e-05 | -35 | -35 | -19 | Fusiform_L |
|  | GI | Negative ^b^ | 97 | 0.110 | 0.070 | - 0.57 | 4.76 | 0.032 | 9.7510e-06 | 19 | -70 | 30 | Cuneus-Precuneus_R |
| APPETITE | N.S. | | | | | | | | | | | | |
| SLEEP | CT | Negative ^b^ | 98 | 0.089 | 0.050 | - 0.55 | 4.83 | 0.032 | 8.4376e-06 | -38 | -33 | 37 | Parietal_Inf_L |
|  | SD | Negative ^b,c^ | 77 | 0.206 | 0.160 | - 0.53 | 4.59 | 0.049 | 1.8569e-05 | 57 | 0 | 40 | Precentral R |
|  |  |  | 144 | 0.046 | 0.033 | - 0.51 | 4.35 | 0.093 | 3.9923e-05 | -49 | -27 | 54 | Postcentral L |
| OVERWHELMED | CT | Positive | 76 | 0.168 | 0.100 | 0.51 | 4.56 | 0.063 | 1.8895e-05 | -5 | 40 | 42 | Frontal_Sup_Medial_L |
| PHYSICAL | SD | Negative | 121 | 0.078 | 0.056 | - 0.48 | 3.96 | 0.235 | 1.3074e-04 | -50 | 4 | -24 | Temporal_Mid_L |
|  | GI | Positive | 88 | 0.139 | 0.090 | 0.50 | 4.43 | 0.081 | 2.8617e-05 | -31 | 13 | 9 | Insula_L |

**Table S2: Whole brain voxel-wise associations between severity of PMDD symptoms and surface measures.** Results from partial correlation analyses ran in SPM using the multiple regression module to assess the relationship between brain structural measures and DRSP scores. Results were visualized at a threshold of p<0.001 uncorrected for multiple testing; however, the FWE corrected statistics for peaks and clusters are reported. DRSP scores corresponding to PMDD symptoms as defined by the DSM-V domains are indicated in capital letters. DRSP, Daily Record of Severity of Problems; CT, cortical thickness; GI, gyrification index; SD, sulcal depth; FWE, Family Wise Error; r, correlation coefficient within the cluster. N.S., non-significant. Secondary local maxima within the significant clusters are not listed.

1. significant after correcting for the number of tests (p_FWE_<0.012 for core symptoms and p_FWE_ <0.007 for secondary symptoms).
2. p<0.05 FWE at the peak voxel level.
3. p<0.05 FWE at the cluster level

| **DRSP scores and items** | **Region** | **Direction of effect** | **r value** | **Statistics**  **95% C.I** | **p value** |
| --- | --- | --- | --- | --- | --- |
| **TOTAL DRSP** | Amygdala | Negative | -0.35 | [-0.57, -0.08] | 0.014 |
| **Core symptoms** |  |  |  |  |  |
| AFFECTIVE LABILITY | Amygdala | Negative | -0.33 | [-0.55, -0.06] | 0.024 |
| IRRITABILITY |  |  | N.S. |  |  |
| DEPRESSION | Amygdala | Negative | -0.39 | [-0.60, -0.13] | 0.006^a^ |
| ANXIETY |  |  | N.S. |  |  |
| **Secondary symptoms** |  |  |  |  |  |
| ANHEDONIA | Amygdala | Negative | -0.29 | [-0.52, -0.02] | 0.042 |
| CONCENTRATION | Amygdala | Negative | -0.29 | [-0.52, -0.02] | 0.050 |
| ENERGY LOSS | Amygdala R | Negative | -0.29 | [-0.52, -0.02] | 0.048 |
| APPETITE | Hippocampus R | Negative | -0.32 | [-0.54, -0.05] | 0.025 |
| SLEEP | Amygdala | Negative | -0.33 | [-0.56, -0.05] | 0.024 |
| OVERWHELMED | Amygdala | Negative | -0.30 | [-0.53, -0.03] | 0.040 |
| PHYSICAL |  |  | N.S. |  |  |

**Table S3: Associations between mean GMV within cortical and subcortical ROIs and severity of PMDD symptoms.** Results from partial correlation analyses assessing the relationship between mean GMV within cortical and subcortical ROIs and DRSP scores. DRSP scores corresponding to PMDD symptoms as defined by the DSM-V domains are indicated in capital letters. C.I., Confidence Interval; DRSP, Daily Record of Severity of Problems; R, right.

^a^ significant after correcting for the number of tests (p_Bonferroni_<0.012 for core symptoms and p_Bonferroni_ <0.007 for secondary symptoms).

| **DRSP scores and items** | **Region** | | **Surface measure** | | **Direction of effect** | | **r-value** | | **Statistics 95% C.I** | **p-value** | |
| --- | --- | --- | --- | --- | --- | --- | --- | --- | --- | --- | --- |
| **TOTAL DRSP** | Caudal ACC L | | Gyrification index | | Positive | | 0.35 | | [0.08, 0.57] | 0.016 | |
|  | Lateral OFC R | | Cortical complexity | | Negative | | -0.30 | | [-0.53, -0.03] | 0.038 | |
|  | PHG L | | Sulcal depth | | Positive | | 0.39 | | [0.13, 0.60] | 0.006*^a^* | |
|  | PHG R | | Cortical complexity | | Negative | | -0.30 | | [-0.53, -0.03] | 0.035 | |
|  | Pars triangularis | | Sulcal depth | | Negative | | -0.28 | | [-0.51, 0.0] | 0.049 | |
| **Core symptoms** |  | |  | |  | |  | |  |  | |
| AFFECTIVE LABILITY | Caudal MFG | | Sulcal depth | | Negative | | -0.29 | | [-0.52, -0.02] | 0.042 | |
|  | *Lateral OFC R* | | *Cortical complexity* | | *Negative* | | *-0.28* | | [-0.51, 0.0] | *0.057* | |
|  | Medial OFC | | Sulcal depth | | Negative | | -0.30 | | [-0.53, -0.03] | 0.038 | |
|  | PHG L | | Sulcal depth | | Positive | | 0.41 | | [0.15, 0.61] | 0.004*^a^* | |
|  | PHG R | | Cortical complexity | | Negative | | -0.31 | | [-0.54, -0.04] | 0.030 | |
|  | *Pars triangularis* | | *Sulcal depth* | | *Negative* | | *-0.27* | | [-0.50, 0.01] | *0.059* | |
|  | SFG | | Sulcal depth | | Negative | | -0.30 | | [-0.53, -0.03] | 0.037 | |
| IRRITABILITY | Caudal ACC L | | *Cortical thickness* | | *Negative* | | *-0.28* | | [-0.51, 0.0] | *0.057* | |
|  | Caudal ACC L | | Gyrification index | | Positive | | 0.33 | | [0.06, 0.55] | 0.020 | |
|  | *Medial OFC* | | *Sulcal depth* | | *Negative* | | *-0.28* | | [-0.51, 0.0] | *0.052* | |
|  | PHG L | | Sulcal depth | | Positive | | 0.37 | | [0.10, 0.58] | 0.009*^a^* | |
|  | PHG R | | Cortical complexity | | Negative | | -0.36 | | [-0.57, -0.09] | 0.012*^a^* | |
|  | Pars triangularis | | Sulcal depth | | Negative | | -0.35 | | [-0.57, -0.08] | 0.014 | |
|  | Rostral MFG R | | Sulcal depth | | Negative | | -0.32 | | [-0.54, -0.05] | 0.027 | |
|  | SFG | | Sulcal depth | | Negative | | -0.33 | | [-0.55, -0.06] | 0.022 | |
| DEPRESSION | Lateral OFC L | | Cortical complexity | | Positive | | 0.29 | | [0.02, 0.52] | 0.046 | |
|  | Lateral OFC R | | Cortical complexity | | Negative | | -0.43 | | [-0.63, -0.17] | 0.002*^a^* | |
|  | PHG L | | Sulcal depth | | Positive | | 0.32 | | [0.05, 0.54] | 0.028 | |
|  | Pars triangularis | | Sulcal depth | | Negative | | -0.29 | | [-0.52, -0.02] | 0.046 | |
| ANXIETY | *Caudal ACC L* | | *Gyrification index* | | *Positive* | | *0.28* | | [0.00, 0.51] | *0.051* | |
|  | Caudal MFG R | | Sulcal depth | | Negative | | -0.29 | | [-0.52, -0.02] | 0.047 | |
|  | Medial OFC L | | Cortical thickness | | Positive | | 0.29 | | [0.02, 0.52] | 0.042 | |
|  | Rostral MFG | | Cortical complexity | | Positive | | 0.33 | | [0.06, 0.55] | 0.021 | |
|  | *SFG R* | | *Cortical thickness* | | *Positive* | | *0.28* | | [0.00, 0.51] | *0.051* | |
| **Secondary symptoms** |  | |  | |  | |  | |  |  | |
| ANHEDONIA | Caudal ACC L | | Gyrification index | | Positive | | 0.31 | | [0.04, 0.54] | 0.032 | |
|  | Lateral OFC R | | Cortical complexity | | Negative | | -0.36 | | [-0.57, -0.09] | 0.011 | |
|  | PHG L | | Sulcal depth | | Positive | | 0.35 | | [0.08, 0.57] | 0.016 | |
|  | Pars Opercularis L | | Gyrification index | | Negative | | -0.33 | | [-0.55, -0.06] | 0.021 | |
|  | Pars Opercularis | | Cortical complexity | | Positive | | 0.30 | | [0.03, 0.53] | 0.036 | |
|  | Pars triangularis | | Sulcal depth | | Negative | | -0.37 | | [-0.58, -0.10] | 0.004*^a^* | |
|  | Rostral MFG | | Gyrification index | | Negative | | -0.32 | | [-0.54, -0.05] | 0.027 | |
|  | *SFG R* | | *Cortical thickness* | | *Positive* | | *0.28* | | [0.00, 0.51] | *0.057* | |
| CONCENTRATION | Caudal MFG R | | Gyrification index | | Negative | | -0.35 | | [-0.57, -0.08] | 0.013 | |
|  | Lateral OFC R | | Cortical complexity | | Negative | | -0.38 | | [-0.59, -0.12] | 0.008 | |
|  | PHG L | | Sulcal depth | | Positive | | 0.34 | | [0.07, 0.56] | 0.017 | |
|  | PHG L | | *Cortical complexity* | | *Negative* | | *-0.28* | | [-0.51, 0.0] | *0.053* | |
|  | Pars opercularis L | | Gyrification index | | Negative | | -0.29 | | [-0.52, -0.02] | 0.045 | |
|  | *Pars opercularis R* | | *Sulcal depth* | | *Positive* | | *0.28* | | [0.00, 0.51] | *0.054* | |
|  | Pars opercularis | | Cortical complexity | | Positive | | 0.35 | | [0.08, 0.57] | 0.015 | |
|  | Rostral ACC R | | Cortical thickness | | Negative | | -0.34 | | [-0.56, -0.07] | 0.017 | |
|  | Rostral MFG | | Gyrification index | | Negative | | -0.30 | | [-0.53, -0.03] | 0.039 | |
| ENERGY LOSS | | Lateral OFC R | | Cortical complexity | | Negative | | 0.30 | [0.03, 0.53] | | 0.036 |
|  | | PHG L | | Sulcal depth | | Positive | | 0.40 | [0.14, 0.60] | | 0.004*^a^* |
|  | | Pars opercularis | | Cortical complexity | | Positive | | 0.34 | [0.07, 0.56] | | 0.018 |
|  | | Pars opercularis L | | Gyrification index | | Negative | | -0.39 | [-0.60, -0.13] | | 0.006*^a^* |
|  | | Pars triangularis | | Sulcal depth | | Negative | | -0.31 | [-0.54, -0.04] | | 0.031 |
|  | | Rostral ACC R | | Cortical thickness | | Negative | | -0.35 | [-0.57, -0.08] | | 0.015 |
|  | |  | | Cortical complexity | | Negative | | -0.29 | [-0.52, -0.02] | | 0.045 |
|  | | Rostral MFG | | Gyrification index | | Negative | | -0.28 | [-0.51, 0.0] | | 0.023 |
| APPETITE | | Insula | | Gyrification index | | Positive | | 0.33 | [0.06, 0.55] | | 0.020 |
|  | | PHG | | Cortical thickness | | Negative | | -0.35 | [-0.57, -0.08] | | 0.016 |
|  | | *PHG R* | | *Gyrification index* | | *Positive* | | *0.27* | [-0.01, 0.50] | | *0.060* |
|  | | PHG R | | Sulcal depth | | Positive | | 0.38 | [0.12, 0.59] | | 0.038 |
|  | | PHG R | | Cortical complexity | | Negative | | -0.37 | [-0.58, -0.10] | | 0.010 |
|  | | Pars Opercularis R | | Gyrification index | | Positive | | 0.30 | [0.03, 0.53] | | 0.037 |
|  | | Rostral ACC L | | Sulcal depth | | Positive | | 0.31 | [0.04, 0.54] | | 0.030 |
|  | | *Rostral ACC R* | | *Cortical thickness* | | *Negative* | | *-0.28* | [-0.51, 0.0] | | *0.055* |
| SLEEP | | Caudal ACC R | | Gyrification index | | Negative | | -0.30 | [-0.53, -0.02] | | 0.042 |
|  | | Caudal MFG R | | Gyrification index | | Negative | | -0.40 | [-0.61, -0.13] | | 0.006*^a^* |
|  | | PHG L | | Sulcal depth | | Positive | | 0.31 | [0.03, 0.54] | | 0.035 |
|  | | Rostral ACC L | | Gyrification index | | Negative | | -0.39 | [-0.60, -0.12] | | 0.007*^a^* |
|  | | SFG | | Cortical thickness | | Positive | | 0.29 | [0.01, 0.52] | | 0.046 |
| OVERWHELMED | | Caudal ACC L | | Gyrification index | | Positive | | 0.30 | [0.03, 0.53] | | 0.038 |
|  | | Caudal MFG R | | Cortical thickness | | Positive | | 0.32 | [0.05, 0.54] | | 0.029 |
|  | | Lateral OFC R | | Cortical complexity | | Negative | | -0.29 | [-0.52, -0.02] | | 0.048 |
| PHYSICAL | | Caudal ACC L | | Gyrification index | | Positive | | 0.37 | [0.10, 0.58] | | 0.009 |
|  | | *Insula* | | *Gyrification index* | | *Positive* | | *0.28* | [0.00, 0.51] | | *0.051* |
|  | | Medial OFC R | | Gyrification index | | Positive | | 0.42 | [0.08, 0.57] | | 0.003*^a^* |
|  | | Medial OFC R | | Cortical complexity | | Negative | | -0.37 | [-0.58, -0.10] | | 0.009 |
|  | | Pars orbitalis L | | Gyrification index | | Negative | | -0.32 | [-0.54, -0.05] | | 0.025 |
|  | | Pars opercularis | | Cortical complexity | | Positive | | 0.30 | [0.03, 0.53] | | 0.040 |
|  | | Rostral ACC L | | Sulcal depth | | Positive | | 0.31 | [0.04, 0.54] | | 0.033 |
|  | | Rostral ACC R | | Cortical thickness | | Negative | | -0.32 | [-0.54, -0.05] | | 0.024 |

**Table S4: Associations between mean surface measures within ROIs and severity of PMDD symptoms.**

Results from partial correlation analyses assessing the relationship between brain surface measures within ROIs and DRSP scores. DRSP scores corresponding to PMDD symptoms as defined by the DSM-V domains are indicated in capital letters. Trend-level correlations defined as 0.05 ≤ p ≤ 0.06 are reported in italic font. ACC, anterior cingulate cortex; C.I., Confidence Interval; DRSP, Daily Record of Severity of Problems; MFG, middle frontal gyrus; OFC, orbitofrontal cortex; PHG, parahippocampal gyrus; SFG, superior frontal gyrus. ^a^ significant after correcting for the number of tests (p_Bonferroni_<0.012 for core symptoms and p_Bonferroni_ <0.007 for secondary symptoms).


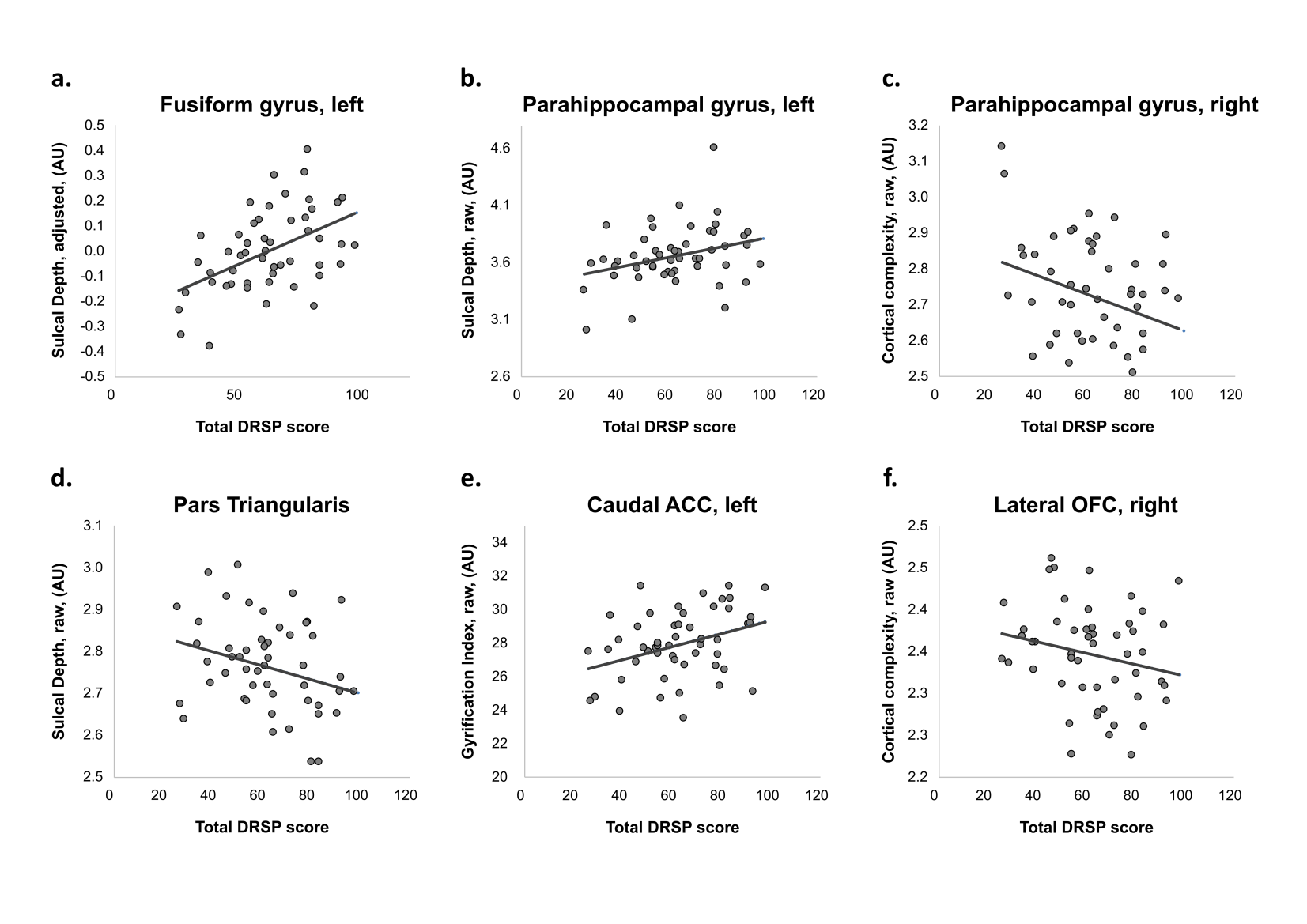


**Figure S1: Correlation plots of surface measures and the total DRSP score.** The graphs illustrate the significant correlations observed between the total DRSP score and surface measures. **a.** scatter plot of the total DRSP score by the average sulcal depth within the left fusiform cluster obtained from the significant whole-brain vertex-wise results (p_FWE_<0.05, corrected for the number of symptoms tested) and adjusted for TIV, age and BMI. **b.** – **f.** scatter plots of the total DRSP score by the raw average surface measures within ROIs obtained from the DKT-40 atlas, unadjusted for covariates (p<0.05, uncorrected for multiple testing). ACC, anterior cingulate cortex; AU, arbitrary unit; DRSP, Daily Record of Severity of Problems; OFC, orbitofrontal cortex.


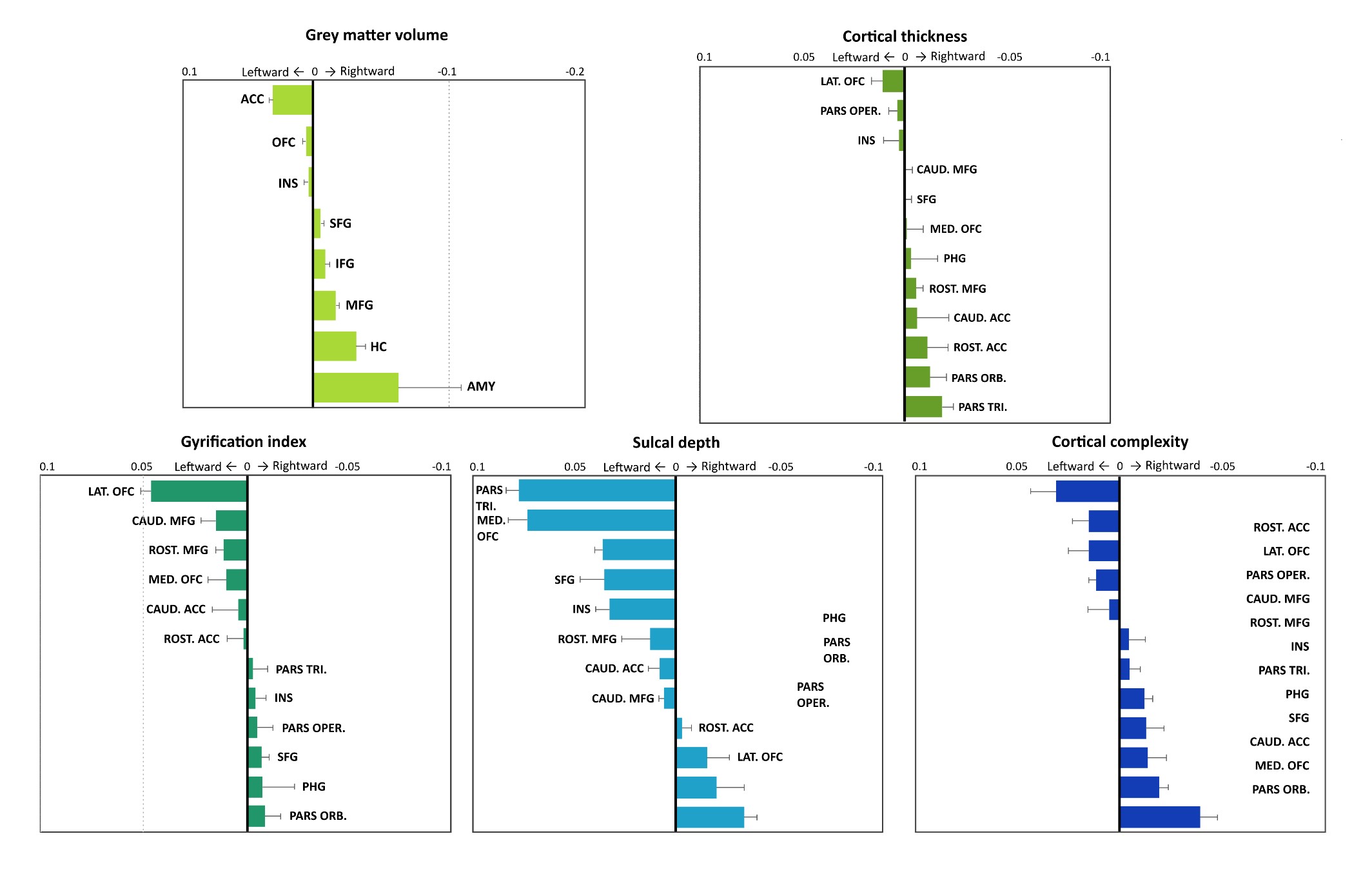


**Figure S2: Asymmetry index of structural brain measures within ROIs.**  ACC, anterior cingulate cortex; CAUD, caudal; DRSP, Daily Record of Severity of Problems; L, left; LAT, lateral; MED, medial; MFG, middle frontal gyrus; OFC, orbitofrontal cortex; PARS ORB, pars orbitalis; PARS OPER, pars opercularis; PARS TRI, pars triangularis; PHG, parahippocampal gyrus; R, right; ROST, rostral; SFG, superior frontal gyrus. Errors bars indicate the standard error of the mean.

**Detailed description of the results**

# Whole-brain grey matter correlates of PMDD symptoms (Figure 2, Table S1)

Among the core PMDD symptoms, affective lability correlated negatively with the left inferior temporal gyrus (ITG) cortical complexity. Conversely, the score of irritability was positively associated with sulcal depth measures in the left PCC. In addition, the score of depression was negatively correlated to the gyrification index of the right PrG.

The severity of secondary symptoms such as anhedonia and energy loss correlated negatively with the gyrification index of the right precuneus-cuneus region. Conversely, positive associations were found between the sulcal depth of the left FuG and the concentration and energy loss scores. Furthermore, negative correlations were found between the sleep DRSP score and both the cortical thickness of the left inferior parietal lobule and the sulcal depth of the PrG and postcentral gyri. Additional trend-level correlations found between surface measures and the DRSP scores are presented in Table S1.

# ROI-based grey matter correlates of PMDD symptoms (Figure 3-4, Table S2-S3)

## Amygdala

The correlation analyses conducted over the subcortical ROIs volumetric measures primarily point to a negative relationship between the bilateral amygdala GMV and the total DRSP score (Figure 3, Table S2).

*Core affective symptoms.* Likewise, GMV in the bilateral amygdala correlated negatively with the severity of core PMDD symptoms as assessed by the affective lability and depression scores (*p_Bonferroni_* < 0.05). *Secondary symptoms.* Anhedonia correlated negatively with GMV of the bilateral amygdala (Table S2). In addition, GMV was negatively associated with concentration, sleep and overwhelmed symptoms in the bilateral amygdala, and energy loss in the right amygdala.

## Anterior Cingulate Cortex

Although no association between ACC GMV and PMDD symptom severity was noted, we found several significant correlations between the ACC surface measures and DRSP scores. Notably, the gyrification index of the left caudal ACC correlated positively with the total DRSP score (Figure 4 and S1, Table S3).

*Core affective symptoms.* The gyrification index of the left caudal ACC was positively associated with irritability, and anxiety at trend level (p=0.051).

*Secondary symptoms.* Surface measures of the ACC were significantly correlated with several secondary symptoms. More specifically, measures of cortical thickness and cortical complexity within the right rostral ACC correlated negatively with symptom severity, while positive correlations were observed for measures of sulcal depth in the left rostral ACC and gyrification of the left caudal ACC, with the exception of sleep that showed negative correlations with gyrification index measures in the left rostral (*p_Bonferroni_* < 0.05) and right caudal ACC.

## Cerebellum (vermis)

Analyses of GMV conducted over the cerebellar vermis did not reveal any significant association with DRSP scores.

## Hippocampus

No significant associations between hippocampus GMV and the severity of PMDD core affective symptoms were noted.

*Secondary symptoms.* Negative correlations were found between the severity of PMDD secondary symptoms and the subcortical ROIs volumetric measures, indicating a negative association between GMV in the right hippocampus and appetite (Figure 3, Table S2).

## Insula

No associations between the severity of PMDD core affective symptoms and insula GMV and surface parameters were noted.

*Secondary symptoms.* Brain surface analyses revealed positive correlations between the gyrification index of the bilateral insula and appetite (Figure 4, Table S3). A similar relationship was observed for the physical DRSP score, at trend level (*p* = 0.051). Analyses of GMV conducted over the insula did not yield any significant results.

## Parahippocampal gyrus

PHG surface measures were associated with various core affective and secondary symptoms (Figure 4). Notably, sulcal depth in the left PHG was positively correlated with the total DRSP score (*p_Bonferroni_* < 0.05), while a negative association was found for cortical complexity in the right PHG (Figure S1, Table S3).

*Core affective symptoms.* Except for anxiety, each of the core PMDD symptoms was positively correlated to the sulcal depth of the left PHG (*p_Bonferroni_* < 0.05 for irritability and affective lability). Conversely, cortical complexity in the right PHG correlated negatively with affective lability and irritability (*p_Bonferroni_* < 0.05).

*Secondary symptoms.* Similar patterns emerged from the analyses of surface measures of the PHG and secondary symptoms, indicating positive relationships between the sulcal depth of the left PHG and anhedonia, concentration, energy loss (*p_Bonferroni_* < 0.05) and sleep, and negative relationships between the cortical complexity of the right PHG and appetite. Likewise, cortical thickness of the bilateral PHG showed negative correlations with appetite.

## Prefrontal cortex

Correlation analyses conducted over the prefrontal surface ROIs and PMDD symptom severity revealed the largest number of associations (Figure 4), whereas analyses of GMV conducted over the PFC ROIs did not reveal any significant correlation (Figure 3). Notably, the total DRSP score correlated negatively with the cortical complexity of the right lateral orbitofrontal cortex (OFC) and the sulcal depth of the bilateral pars triangularis region of the inferior frontal gyrus (IFG) (Figure S1, Table S3).

*Core affective symptoms.* Affective lability correlated negatively with the sulcal depth of the bilateral superior frontal gyrus (SFG), caudal MFG and medial OFC. In addition, irritability was negatively correlated with the sulcal depth of the right rostral MFG and bilateral SFG and pars triangularis. Depression also showed a negative association with the sulcal depth of the bilateral pars triangularis, in addition to a lateralized pattern of correlation with the cortical complexity of the lateral OFC, showing negative and positive associations in the right and left hemispheres, respectively. Last, anxiety correlated positively with the cortical thickness of the left medial OFC and the complexity of the bilateral rostral MFG, while negatively with the sulcal depth of the right caudal MFG.

*Secondary symptoms.* Numerous correlations were found between prefrontal surface measures and secondary symptoms, including regions of the superior, middle and inferior frontal gyri, and OFC. Thus, anhedonia and concentration correlated negatively with the surface measures of IFG (*p_Bonferroni_* < 0.05), MFG and lateral OFC ROIs, while positively with the cortical complexity of the pars opercularis. Furthermore, energy loss correlated negatively with the gyrification (*p_Bonferroni_* < 0.05) and sulcal depth measures of IFG regions and rostral MFG, and positively with the complexity of the right lateral OFC and bilateral pars opercularis. Positive correlations were also found between appetite and the gyrification index of the right pars opercularis, while sleep correlated negatively with the gyrification index of the right caudal MFG (*p_Bonferroni_* < 0.05) and positively with the cortical thickness of the bilateral SFG. In addition, the overwhelmed DRSP score was positively correlated with the cortical thickness of right caudal MFG, while negatively correlated with the cortical complexity of the right lateral OFC. The physical DRSP score was positively correlated with gyrification index of the right medial OFC (*p_Bonferroni_* < 0.05) and the complexity of the bilateral pars opercularis, while negatively correlated with the gyrification index of the left pars orbitalis region of the IFG and the complexity of the right medial OFC.

# Detailed discussion of symptoms-wise associations with grey matter structure

Irritability, one of the core symptoms characterizing PMDD, correlated positively with the sulcal depth

of the PHG and PCC, while negatively with PFC regions, and negatively with the gyrification of the caudal ACC in PMDD women. In healthy subjects, irritability relates to thinner cortices in brain regions implicated in emotion regulation, such as the orbitofrontal, lateral temporal, and medial temporal cortex ^1^. Similarly, thinner right ACC was associated with higher irritability in healthy subjects and patients with major depressive disorder ^2^. Conversely, positive correlations were found between irritability and the cortical thickness of the PrG, the gyrification of the left insula, and GMV of the ACC, OFC, lingual and postcentral gyrus ^3^. Although no correlations were found between irritability and cortical thickness or GMV in our sample of women with PMDD, the location of the associations observed with sulcal depth and gyrification measures are in line with the previous neuroimaging studies on irritability.

Depression is also a key symptom of PMDD, which correlated negatively with the GMV of the amygdala, in our sample. In contrast, recent associations between brain structure and depressive symptoms in subclinical populations, mainly point to a negative correlation between depressed mood and GMV of the hippocampus, as well as the ACC, where the effect is more prominent in women ^4^. Additionally, relationships between GMV and depressive symptoms were shown in the PHG, ITG and PFC across the depressive spectrum including healthy subjects and MDD patients, although the direction of effect in frontal regions seems less consistent ^2,5^. In line with this, we observed both positive and negative correlations between depression and surface measures in the PHG and several frontal ROIs in women with PMDD. Interestingly, the severity of anhedonia, which is closely related to the depressive symptomatology, displayed similar associations, involving GMV of the amygdala and surface measures of the lateral OFC, IFG sub-regions and PHG.

Affective lability, another core symptom of PMDD, is shared with the diagnosis of borderline

personality disorder. Studies on borderline personality disorder showed positive correlations between the severity of affective lability and the reactivity of the amygdala and PHG during emotion processing tasks ^6,7^, along with a negative correlation between emotion regulation and IFG reactivity during trials assessing regulatory ability in women ^7^. In women with PMDD, we found negative associations between affective lability and GMV of the amygdala, as well as surface parameters of the medial OFC, MFG, SFG, ITG and PHG. While evidence of the neuroanatomical correlates of affective lability is scarce, the previous functional findings and the present results overlap with the models of mood regulation and the emotional brain, both involving the amygdala and PFC ^8,9^.

Anxiety, the fourth core symptom of PMDD, was positively correlated with the medial OFC thickness,

and cortical complexity of the rostral MFG, but negatively correlated with the sulcal depth of the caudal MFG. In healthy women with sub-clinical anxiety symptoms, associations were found between symptom severity and GMV of the amygdala, ACC, insula, OFC, hippocampus and PHG, although the direction of these relationships is somewhat inconsistent ^4^. Likewise, findings from studies on anxiety disorders mainly point to positive correlations between anxiety scores and GMV in frontal, cingulate and insular regions ^5,10-12^, albeit a negative association with the medial PFC volume was reported as well ^13^.

# References

1. Jirsaraie, R. J. *et al.* Accelerated cortical thinning within structural brain networks is associated with irritability in youth. *Neuropsychopharmacology* **44**, 2254-2262, doi:10.1038/s41386-019-0508-3 (2019).
2. Lener, M. S. *et al.* Cortical abnormalities and association with symptom dimensions across the depressive spectrum. *J Affect Disord* **190**, 529-536, doi:10.1016/j.jad.2015.10.027 (2016).
3. Besteher, B. *et al.* Brain structural correlates of irritability: Findings in a large healthy cohort. *Hum Brain Mapp* **38**, 6230-6238, doi:10.1002/hbm.23824 (2017).
4. Besteher, B., Gaser, C. & Nenadic, I. Brain Structure and Subclinical Symptoms: A Dimensional Perspective of Psychopathology in the Depression and Anxiety Spectrum. *Neuropsychobiology* **79**, 270-283, doi:10.1159/000501024 (2020).
5. Qi, H. *et al.* Gray matter volume abnormalities in depressive patients with and without anxiety disorders. *Medicine (Baltimore)* **93**, e345, doi:10.1097/MD.0000000000000345 (2014).
6. Perez, D. L. *et al.* Frontolimbic neural circuit changes in emotional processing and inhibitory control associated with clinical improvement following transference-focused psychotherapy in borderline personality disorder. *Psychiatry Clin Neurosci* **70**, 5161, doi:10.1111/pcn.12357 (2016).
7. Silvers, J. A. *et al.* Affective lability and difficulties with regulation are differentially associated with amygdala and prefrontal response in women with Borderline Personality Disorder. *Psychiatry Res Neuroimaging* **254**, 74-82, doi:10.1016/j.pscychresns.2016.06.009 (2016).
8. Davey, C. G. *et al.* Functional brain-imaging correlates of negative affectivity and the onset of first-episode depression. *Psychol Med* **45**, 1001-1009, doi:10.1017/S0033291714002001 (2015).
9. Pessoa, L. A Network Model of the Emotional Brain. *Trends Cogn Sci* **21**, 357-371, doi:10.1016/j.tics.2017.03.002 (2017).
10. Schienle, A., Ebner, F. & Schafer, A. Localized gray matter volume abnormalities in generalized anxiety disorder. *Eur Arch Psychiatry Clin Neurosci* **261**, 303-307, doi:10.1007/s00406-010-0147-5 (2011).
11. Moon, C. M., Kim, G. W. & Jeong, G. W. Whole-brain gray matter volume abnormalities in patients with generalized anxiety disorder: voxel-based morphometry. *Neuroreport* **25**, 184-189, doi:10.1097/WNR.0000000000000100 (2014).
12. Ma, Z. *et al.* Frontoparietal network abnormalities of gray matter volume and functional connectivity in patients with generalized anxiety disorder. *Psychiatry Res Neuroimaging* **286**, 24-30, doi:10.1016/j.pscychresns.2019.03.001 (2019).
13. Kim, G. W., Yoon, W. & Jeong, G. W. Whole-brain volume alteration and its correlation with anxiety severity in patients with obsessive-compulsive disorder and generalized anxiety disorder. *Clin Imaging* **50**, 164-170, doi:10.1016/j.clinimag.2018.03.008 (2018).
